# Proteolytic Activity in Meadow Soil after the Application of Phytohormones

**DOI:** 10.3390/biom9090507

**Published:** 2019-09-19

**Authors:** Ladislav Holik, Valerie Vranová

**Affiliations:** Department of Geology and Pedology, Faculty of Forestry and Wood Technology, Mendel University in Brno, 613 00 Zemědělská 1, Czech Republic; valerie.vranova@mendelu.cz

**Keywords:** auxins, cytokinins, plant growth-promoting rhizobacteria, soil protease

## Abstract

Phytohormones, similar to soil enzymes, are synthesized and secreted into the soil environment by fungi and microorganisms. Phytohormones are involved in regulating microbial community activity in the rhizosphere. This paper examines how auxins, cytokinins, ethephon and chlorocholine chloride affect the activity of native soil proteases in the organo-mineral horizon of an alpine meadow. In the meadow habitat, native soil proteases were inhibited by auxins whereas the effect of cytokinins on these enzymes was not statistically significant. A similar inhibitory effect on the activity of proteases was shown for ethephon and chlorocholine chloride, both of which also inhibited the activity of native soil proteases in the alpine meadow soil. Overall, the inhibitory effect of phytohormones on the activity of native protease activity may affect plant nutrition by retarding the nitrogen cycle in the soil. This work contributes to our understanding of the influence of substances produced by the rhizosphere that can actively participate in the activity of soil microorganisms and consequently influence the soil nitrogen cycle.

## 1. Introduction

One of the most important natural resources is the soil, on which plants, animals and micro-organisms are directly or indirectly dependent [1,2]. The decomposition of organic matter produced by plants, animals and microorganisms is an important ecological process that ensures bioavailable forms of nutrients bound to it, and is mediated in the soil by extracellular enzymes. These enzymes are produced by plants and microorganisms [3]. One of these enzymes is a protease that mediates hydrolysis of the protein part of the organically bound nitrogen into single amino acids [4]. Proteolysis is therefore an important process in the nitrogen cycle and is considered as a limiting factor in soil nitrogen mineralization [5].

A diverse array of organisms in the soil play a key role in pedogenesis and nutrient cycling through various interactions [1,2]. Microorganisms interact with plants, directly affecting their growth [6] and are beneficial for the overall health of plants. Plant growth-promoting rhizobacteria (PGPRs) are located close to the plant root [7,8] and PGPRs in the rhizosphere favourably impact plant growth by direct and indirect mechanisms that increase productivity and control pathogenic microorganisms. PGPRs promote plant growth by producing substances useful for their growth, such as phytohormones, or by promoting the absorption of nutrients (iron, phosphorus and others). These mechanisms include nitrogen fixation, plant growth regulation, enhanced phosphorus solubility and siderophore production [9,10].

This work was conducted to determine the effect of auxins, cytokinins, ethephon and chlorocholine chloride on the native protease activity of alpine meadow soil. Our hypothesis was that auxins would increase the soil protease activity, and cytokinins, ethephon and chlorocholine chloride would inhibit the activity of proteases. We tested two naturally occurring auxins (indole-3-acetic acid and indole-3-butyric acid), two synthetic auxins (1-naphthaleneacetic acid and 2-naphthoxyacetic acid), and two cytokinins (6-benzylaminopurine and adenine hemisulphate) within the study. Ethephon, which is an ethylene-releasing compound [11], and chlorocholine chloride, used as a plant growth regulator, were also included [12].

## 2. Materials and Methods

Soil samples for the experiments were collected from the Bílý Kříž experimental ecological site (part of the European infrastructure of the Project ICOS (Integrated Carbon Observation System) incorporated in the international research infrastructure ESFRI (European Strategy Forum on Research Infrastructures), located in the Moravian-Silesian Beskids in the north-eastern part of the Czech Republic (N 49°30′17″, E 18°32′28″), with an altitude of 825-860 m above the sea level, an average annual temperature of 6.7 °C and annual rainfall of 1239 mm. The experimental area was a mountain meadow with a haplic Stagnosol soil type [13]. The soil samples were taken from the organo-mineral horizon (Ah horizon). Three random samples where composited and passed through a 2 mm mesh sieve. Table 1 shows selected physical and chemical properties of the test soil.

The activity of native soil proteases was determined spectrophotometrically according to the methodology of Rejsek et al. [14]: 100 μL of distilled water with dissolved PGR at 0, 5, 50 and 100 μg per gram of dry soil was added to 2 grams of the soil sample and incubated for 2 hours. The experiment included naturally occurring auxins indole-3-acetic acid (IAA), indole-3-butyric acid (IBA), two synthetic auxins 1-naphthaleneacetic acid (NAA) and 2-naphthoxyacetic acid (NOA), two cytokinins 6-benzylaminopurine (BA) and adenine hemisulphate (AH) and two PGRs, ethephon (ET) and chlorocholine chloride (CCC). Auxins were selected based on similarity to the effect of IAA on microorganisms and plants. IBA is an IAA precursor (or may be an auxin according to some authors).

NAA and NOA are synthetic auxins and their influence on cells is similar to that of IAAs. ET and CCC were selected for their indirect effect on cells (e.g. initiation of ethylene production). The soil pH was measured in a soil:water suspension with a ratio of 1:2.5 in 1 M KCl. Total carbon (Ct) and total nitrogen (Nt) were determined on LECO TruSpec Analyzer (LECO, St. Joseph, MI, USA), calibrated to LECO standards: Tobacco 1016.

Statistical evaluation of the results of the native protease activity of the individual samples was performed using single factor ANOVA and multiple comparison of HSD by means of the Tukey test in Statistica 13.3 (TIBCO Software Inc., Palo Alto, CA, USA).

## 3. Results and Discussion

The auxins examined inhibited the native soil protease activity at the meadow site (Table 2). The auxin IBA had the greatest impact, reducing the activity of native proteases from 78.49 μg L-tyrosine (0 μg IBA) to 48.45 μg L-tyrosine (100 μg IBA). Significant inhibition of native proteases occurred in response to the IAA and NOA auxins, both of which led to a similarly large activity reduction at 50 and 100 μg (IAA and NOA) (Table 2). The smallest inhibition of the native protease activity was achieved with the synthetic auxin NAA and only at 50 μg NAA.

Auxins influence plant root development and architecture, such as primary root growth, lateral root formation, and the development of root hairs [15]. Two synthetic auxins (NAA, NOA) and two naturally occurring auxins (IAA, IBA) were examined in our study. Auxins had a negative effect on the soil protease activity. Synthetic auxins also have inhibitory effects on root growth [16]. Gómez and Carpena [17] identified that these auxins stimulated root exudation and inhibited root growth. The auxin IBA may be a precursor to IAA formation or occur as a storage form of auxin, enabling spatial and time control of the level of the auxin indole-3-acetic acid in the soil environment [18]. Liu et al. [19] and Goswami et al. [8] suggest IBA may also be synthesized by soil microorganisms, not only by plants. There is little information about the effect of IBA on microorganisms in the rhizosphere. However, we found in our previous study [20] that auxins stimulated native protease activity in the organo-mineral horizon. Bacterial IAA stimulates plant root growth [21], root dilation and increases the number and length of primary and lateral roots [22]. At the meadow site, however, we found that the presence of IAA inhibited the activity of native proteases. A similar inhibitory effect of IAA has also been found by Luo et al. [23] when examining the response of fungal hyphae to the presence of IAA in culture conditions. As in our case, with increasing amounts of IAA, the fungal mycelia decreased. This fungal reaction is interesting because mushrooms are capable, like bacteria, of producing IAA, as found in the works by Tsavkelova et al. [24], Kulkarni et al. [25] and Krause et al. [26].

The cytokinins, BA and AH, did not have a statistically significant effect (*P* < 0.05) on the native protease activity of the alpine meadow soils. CCC inhibited the activity of native soil proteases by about 10%, and the greatest reduction in L-tyrosine production occurred at 50 μg CCC (Table 2). The inhibition of the native proteolytic activity of soils within ET occurred only at 100 μg ET, and the amount of L-tyrosine produced decreased to 66.86 μg.

Ethephon (ET) is a plant growth regulator [27], which releases ethylene after autohydrolysis and thus promotes the growth of adventitious roots in plants [28]. The application of ET caused the inhibition of native soil proteases at the meadow site. We found where there was inhibition of the native soil proteolytic activity, similar to Holik et al. [29]. The presence of ET had an inhibitory effect on the growth of plants, their photosynthesis and accumulation of nitrogen as reported in Khan et al. [11]. Chlorocholine chloride (Chlormequat), inhibits the synthesis of gibberellin, thus improving root growth and increasing tolerance to water stress. CCC as well as ET have been shown to have an inhibitory effect on the activity of native soil proteases [12].

The NOA results strongly correlated with IBA (0.8070) and IAA (0.9328) at the meadow site (Table 2). A strong positive correlation was identified between IBA and IAA (0.8511). The correlations of the results between the cytokinins BA and CCC (0.6583) as well as between AH and ET (0.6233) were also estimated (Table 2).

Thus, our results indicate a negative effect of auxins on the proteolytic activity of meadow soil and thus a potential retardation of nitrogen mineralization because the protease (and the enzymes as a whole) are key to the availability of nutrients for plants [30]. Since protein depolymerization limits the rate of mineralization of soil organic nitrogen [31], even a slight change may affect the rate of nitrogen mineralization [32]. On the other hand, a decrease in protease activity, and hydrolytic activity overall, may lead to (or also indicate) stabilization of organic matter [33]. However, further experiments are needed to show the importance of the observed effect of phytohormones on soil enzyme activity, as described here for proteases, since other enzymes may react differently to the same soil factors [34].

## 4. Conclusions

Auxins, ethephon and chlorocholine chloride inhibited the native protease activity in mountain meadow soil. Cytokinins did not have a statistically significant impact on the activity of this enzyme. Therefore, our hypothesis was confirmed only in the case of the soil protease activity results after ethephon and chlorocholine chloride addition. This work contributes to the better understanding of the influence of auxins, cytokinins, ethephon and chlorocholine chloride on the activity of soil microorganisms and the availability of organic nitrogen to plants.

## Figures and Tables

**Table 1:** Selected physical and chemical properties of the tested soils.

Plot	C_t_ (%)	N_t_ (%)	C/N	pH H_2_O	pH 1M KCl	Clay (%)	Silt (%)	Sand (%)
Meadow, Ah horizon (haplic Stagnosol)	4.94	0.43	11.5	4.60	3.61	18.60	26.00	55.40

C_t_—total carbon, N_t_—total nitrogen.

**Table 2:** The native proteolytic activity after addition of auxins, cytokinins, ethephon and chlorocholine chloride in amounts from 0, 5, 50 a 100 µg/g dry soil in alpine meadow haplic Stagnosol.

Treatment	0	5	50	100
1-naphthaleneacetic acid	78.49 ± 1.29 ^ab^	81.67 ± 0.21 ^b^	75.32 ± 1.88 ^a^	77.44 ± 0.63 ^ab^
2-naphthoxyacetic acid	78.49 ± 1.29 ^b^	79.55 ± 1.29 ^b^	72.36 ± 1.32 ^a^	68.76 ± 0.92 ^a^
Indole-3-butyric acid	78.49 ± 1.29 ^c^	63.90 ± 1.53 ^b^	53.74 ± 1.18 ^a^	48.45 ± 0.92 ^a^
Indole-3-acetic acid	78.49 ± 1.29 ^a^	77.65 ± 1.29 ^a^	69.40 ± 0.56 ^c^	60.72 ± 1.48 ^b^
6-benzylaminopurine	78.49 ± 1.29	77.86 ± 1.18	74.69 ± 0.92	74.69 ± 1.06
Adenine hemisulfate	78.49 ± 1.29 ^ab^	85.48 ± 2.08 ^a^	83.57 ± 1.88 ^a^	73.20 ± 1.48 ^b^
Ethephon	78.49 ± 1.29 ^a^	81.88 ± 2.57 ^a^	81.03 ± 2.60 ^a^	66.86 ± 2.38 ^b^
Chlorocholine chloride	78.49 ± 1.29 ^b^	70.45 ± 1.27 ^a^	68.97 ± 1.18 ^a^	70.24 ± 2.70 ^a^
Correlation coefficients (*p*)				
Auxin				
1-naphthaleneacetic acid	1.0000			
2-naphthoxyacetic acid	0.5680	1.0000		
Indole-3-butyric acid	0.3673	0.8071 **	1.0000	
Indole-3-acetic acid	0.4902	0.9328 **	0.8511 **	1.0000
Cytokinin				
6-benzylaminopurine	1.0000			
Adenine hemisulfate	0.3481	1.0000		
Ethephon	0.3612	0.6233 **	1.0000	
Chlorocholine chloride	0.6583 **	−0.1495	0.1034	1.0000

Results represent the amount of produced L-tyrosine in μg/g/h dry soil. Means with standard errors of the mean (SE) followed by the same letter (^a, b, c^ horizontally, for each point separately) were not significantly different at the 0.05 probability level. Standard Error SE ± for Tukey HSD post hoc test, n = 3. Statistically significant correlations (*P* < 0.05; n = 12) are designated as **.
